# Effects of Hearing Aid Noise Reduction on Early and Late Cortical Representations of Competing Talkers in Noise

**DOI:** 10.3389/fnins.2021.636060

**Published:** 2021-03-26

**Authors:** Emina Alickovic, Elaine Hoi Ning Ng, Lorenz Fiedler, Sébastien Santurette, Hamish Innes-Brown, Carina Graversen

**Affiliations:** ^1^Eriksholm Research Centre, Oticon A/S, Snekkersten, Denmark; ^2^Department of Electrical Engineering, Linkoping University, Linkoping, Sweden; ^3^Centre for Applied Audiology Research, Oticon A/S, Smørum, Denmark; ^4^Department of Behavioral Sciences and Learning, Linkoping University, Linkoping, Sweden; ^5^Department of Health Technology, Technical University of Denmark, Lyngby, Denmark

**Keywords:** selective auditory attention, hearing aids, electroencephalography (EEG), stimulus reconstruction, hierarchical processing, hearing impairment, noise reduction scheme, cortical speech tracking

## Abstract

**Objectives:**

Previous research using non-invasive (magnetoencephalography, MEG) and invasive (electrocorticography, ECoG) neural recordings has demonstrated the progressive and hierarchical representation and processing of complex multi-talker auditory scenes in the auditory cortex. Early responses (<85 ms) in primary-like areas appear to represent the individual talkers with almost equal fidelity and are independent of attention in normal-hearing (NH) listeners. However, late responses (>85 ms) in higher-order non-primary areas selectively represent the attended talker with significantly higher fidelity than unattended talkers in NH and hearing–impaired (HI) listeners. Motivated by these findings, the objective of this study was to investigate the effect of a noise reduction scheme (NR) in a commercial hearing aid (HA) on the representation of complex multi-talker auditory scenes in distinct hierarchical stages of the auditory cortex by using high-density electroencephalography (EEG).

**Design:**

We addressed this issue by investigating early (<85 ms) and late (>85 ms) EEG responses recorded in 34 HI subjects fitted with HAs. The HA noise reduction (NR) was either on or off while the participants listened to a complex auditory scene. Participants were instructed to attend to one of two simultaneous talkers in the foreground while multi-talker babble noise played in the background (+3 dB SNR). After each trial, a two-choice question about the content of the attended speech was presented.

**Results:**

Using a stimulus reconstruction approach, our results suggest that the attention-related enhancement of neural representations of target and masker talkers located in the foreground, as well as suppression of the background noise in distinct hierarchical stages is significantly affected by the NR scheme. We found that the NR scheme contributed to the enhancement of the foreground and of the entire acoustic scene in the early responses, and that this enhancement was driven by better representation of the target speech. We found that the target talker in HI listeners was selectively represented in late responses. We found that use of the NR scheme resulted in enhanced representations of the target and masker speech in the foreground and a suppressed representation of the noise in the background in late responses. We found a significant effect of EEG time window on the strengths of the cortical representation of the target and masker.

**Conclusion:**

Together, our analyses of the early and late responses obtained from HI listeners support the existing view of hierarchical processing in the auditory cortex. Our findings demonstrate the benefits of a NR scheme on the representation of complex multi-talker auditory scenes in different areas of the auditory cortex in HI listeners.

## Summary

Previous research using non-invasive (magnetoencephalography, MEG) and invasive (electrocorticography, ECoG) neural recordings has demonstrated the progressive and hierarchical representation and processing of complex multi-talker auditory scenes in the auditory cortex. Early responses (<85 ms) in primary-like areas appear to represent the individual talkers with almost equal fidelity and are independent of attention in normal-hearing (NH) listeners. However, late responses (>85 ms) in higher-order non-primary areas selectively represent the attended talker with significantly higher fidelity than unattended talkers in NH and hearing–impaired (HI) listeners. The objective of this study was to investigate the effect of a noise reduction scheme (NR) in a commercial hearing aid (HA) on the representation of complex multi-talker auditory scenes in distinct hierarchical stages of the auditory cortex by using high-density electroencephalography (EEG). We addressed this issue by investigating early and late EEG responses recorded in 34 HI subjects fitted with HAs. The HA noise reduction (NR) was either on or off while the participants listened to a complex auditory scene. Participants were instructed to attend to one of two simultaneous talkers in the foreground while multi-talker babble noise played in the background (+3 dB SNR). We found that the NR scheme contributed to the enhancement of the foreground and of the entire acoustic scene in the early responses, and that this enhancement was driven by better representation of the target speech. We found that the target talker in HI listeners was selectively represented in late responses. Furthermore, we found that use of the NR scheme resulted in enhanced representations of the target and masker speech in the foreground and a suppressed representation of the noise in the background in late responses. Finally, we found a significant effect of late time window on the strengths of the cortical representation of the target and masker, respectively. Together, our analyses of the early and late responses obtained from HI listeners support the existing view of hierarchical processing in the auditory cortex. Our findings demonstrate the benefits of a NR scheme on the representation of multi-talker auditory scenes in different areas of the auditory cortex in HI listeners.

## Introduction

In multi-talker environments, listeners can focus their attention to a certain talker while “tuning out” other interfering talkers at different locations (Cherry, 1953; McDermott, 2009). This ability has been shown to be reflected in neural responses recorded using electroencephalography (EEG), such that responses to the target (attended) talker are selectively enhanced, and neural responses related to the masker (ignored) talkers are suppressed (Mesgarani and Chang, 2012; Zion Golumbic et al., 2013). These results led to the assumption that the ability to selectively attended to one sound source is determined by how well the cortical representation (or tracking) of the target source is enhanced and how well the representation (or tracking) of interfering masker sources is suppressed. This selective attention may partly depend on acoustic cues (e.g., voice fundamental frequency, prosody, spatial locations of talkers, etc.) which facilitate “unmixing” of sounds, i.e., parsing of the incoming signal into distinct internal representations–auditory objects–for each of the talkers (Griffiths and Warren, 2004; Shinn-Cunningham, 2008), so that the listeners are able to selectively attend, i.e., track and understand the speech of a relevant (target) talker. However, the required neural computations underlying “unmixing” of sound sources, the level at which this unmixing occurs, and the role of attention in the process, remain a subject of debate (Shinn-Cunningham et al., 2017).

Previous research has provided evidence for a progressive and hierarchical organization along the auditory pathway from the periphery all the way to primary and higher-order non-primary auditory cortex (Davis and Johnsrude, 2003; Hickok and Poeppel, 2007; Okada et al., 2010; Peelle et al., 2010; Overath et al., 2015). Latencies of neural responses to sound entering the ear progress in serial fashion across different regions of human auditory cortex (Inui et al., 2006; Nourski et al., 2014; Santoro et al., 2014). Because of this serial mode of auditory processing, the hierarchical organization of speech and language processing can be depicted by both underlying anatomy and response latency. The former can be studied using invasive direct cortical (e.g., electrocorticography, ECoG) recordings, whereas the latter can be studied by exploiting the high temporal precision of non-invasive scalp electro- and magnetoencephalography (EEG and MEG) neural recordings.

Recent non-invasive studies on selective attention using natural speech have shown that cortical auditory processing involves at least two major separable neural processing components. Using MEG recordings from listeners with normal hearing thresholds (NH listeners), it has been suggested that ***early*** (<85 ms) components of cortical responses represent (track) the entire acoustic scene. Here, individual source signals (talkers) are represented (tracked) with almost equal fidelity and are largely unaffected by selective attention (Puvvada and Simon, 2017; Brodbeck et al., 2020). Using MEG (Ding and Simon, 2012a, b; Puvvada and Simon, 2017) and EEG (Power et al., 2012; O’Sullivan et al., 2015; Fiedler et al., 2019) recordings from NH listeners, it has been shown that responses representing the target talker are encoded with significantly higher fidelity than for masker talkers in late (>85 ms) components. In addition, previous studies using invasive cortical recordings have suggested that early responses are generated within primary areas of auditory cortex (Heschl’s Gyrus, HG), whereas late responses are generated within higher-order non-primary areas of auditory cortex (superior temporal gyrus, STG) (Mesgarani and Chang, 2012; Zion Golumbic et al., 2013; O’Sullivan et al., 2019). In other words, these studies suggest that, while target and masker talkers are co-represented in early responses in the primary auditory cortex, late responses in higher-order auditory areas further away from primary auditory cortex dynamically change to mainly represent the target talker. Furthermore, several MEG studies have shown that although the ability to track the stimulus envelope in the cortex substantially worsens as the window decreases down to 100 ms (Ding and Simon, 2013), older adults still showed evidence of enhanced stimulus envelope reconstruction (Presacco et al., 2016, 2019).

Hearing impairment, however, can significantly disrupt behavioral performance of selective attention in complex auditory scenes comprising multiple competing talkers (Gatehouse and Noble, 2004). Even when hearing loss (HL) is compensated for using hearing aid (HA) technology, a well-fit HA does not restore all aspects of hearing (Shinn-Cunningham and Best, 2008). Previous studies have reported at least two different processes that could be each is impeded by hearing impairment (Shinn-Cunningham and Best, 2008). First, spectro-temporal details are not encoded robustly in hearing–impaired (HI) listeners. This may lead to impaired formation of individual auditory objects and a degraded representation of the auditory scene, thus making it difficult for HI listeners to perceptually segregate competing sounds (Gaudrain et al., 2007). Second, the degraded representation of the auditory scene may result in an impaired ability to selectively enhance the target talker and suppress other competing masker talkers, thus resulting in impaired selective attention (Dai et al., 2018). Proceeding along, the impaired selective attention in HI listeners may remain despite the assistance of technologies such as of HAs (Marrone et al., 2008; Best et al., 2010).

How impaired selective attention in HI listeners is represented in electrophysiological signals is a matter of current debate. Independent of attention, HI listeners exhibit abnormally enhanced cortical responses to speech (Millman et al., 2017; Goossens et al., 2018; Mirkovic et al., 2019; Presacco et al., 2019; Decruy et al., 2020; Fuglsang et al., 2020). While some studies show that in HI listeners the cortical selectivity between target and masker is impaired (Petersen et al., 2017; Dai et al., 2018), other studies show that the enhanced cortical representation of the target talker compared to the masker talker in the late responses is similar to that seen in NH listeners (Decruy et al., 2020; Fuglsang et al., 2020). Several studies have reported that more accurate cortical tracking of speech is positively correlated with speech understanding ability in both NH (Das et al., 2018; McHaney et al., 2020) and HI listeners (Presacco et al., 2019; Decruy et al., 2020). Moreover, improved cognitive functions and enhanced cortical speech processing were found to be associated with auditory rehabilitation through the use of HAs (Karawani et al., 2018). More specifically, cortical response amplitudes, characterized by positive P1 peak at around 50 ms), negative N1 peak at around 100 ms and positive P2 peak at around 200 ms (Martin et al., 2007), were measured. They reported that although the HA use for a period of 6 months did not affect P1 amplitudes, N1, and P2 amplitudes [markers of sensory memory (Picton, 2013) and auditory learning (Ross et al., 2013)] increased after 6 months of HA use.

In addition, results from our previous study (Alickovic et al., 2020) showed that a noise reduction (NR) scheme available in a commercial HA enhanced cortical representations for the target and masker talkers (which is defined as the foreground) at both low and high SNRs and suppressed the cortical representation of background noise at low SNRs. This suggests that HA signal processing may benefit HI listeners in terms of enhanced contrast between cortical neural representations of the foreground and the background in the scene.

Motivated by these findings, the objective of the current study was to investigate how a NR scheme available in a commercial HA affected the processing of speech at early and late stages of auditory processing in listeners with HL. To test this, we compared the neural representation of a variety of different objects or their combination within a complex auditory scene, by analyzing a new EEG dataset collected from 34 HI listeners with the system-identification method of stimulus reconstruction (SR).

Our main hypothesis was that HI listeners would derive a benefit from the NR scheme in terms of the representation of complex multi-talker acoustic scenes at distinct hierarchical stages of auditory processing. We tested the following hypotheses:

(H1) That cortical representations of the foreground and entire acoustic scene in the early responses (dominated by primary areas) would be more accurate (i.e., more enhanced) with the NR on compared to off.
(H2) Consistent with previous studies using MEG and ECoG in NH listeners, the late EEG responses (dominated by higher-order non-primary areas) in HI listeners selectively represent the target talker with significantly higher fidelity than the masker talker.
(H3) Consistent with our previous findings, the NR scheme enhances the individual representations of the target and masker talkers in the front hemifield and suppresses the representation of the noise in the background in late responses.

We then extended our analysis to include the effect of changing the time window duration of speech-evoked EEG responses on cortical representation of individual talkers and employ it to investigate whether activating the NR scheme reduces the time window required to maximize the stimulus reconstructions.

## Materials and Methods

### Study Population

**FIGURE 1:**
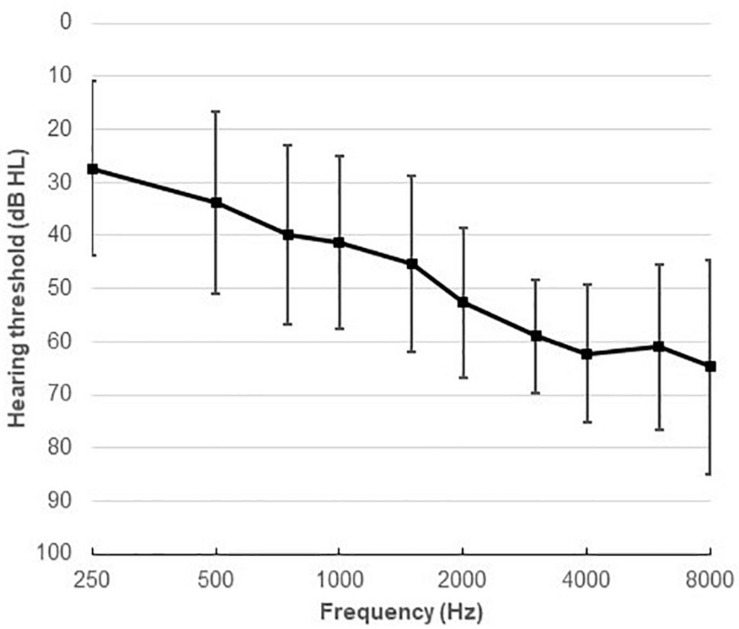
Average audiometric thresholds (mean and SD) at octave and inter-octave frequencies across 250–8,000 Hz.

Thirty-four native Danish speakers (24 males), aged between 21 and 84 years (mean age 64.2, SD 13.6) participated in the study. All participants were fitted binaurally with two HAs. They all had mild to moderately severe symmetrical sensorineural HL, with an average 4-frequency PTA of 47.5 dB HL. Figure 1 shows the average audiogram. All the participants were experienced HA users. All had normal or corrected-to-normal vision, and had no history of neurological disorders, dyslexia, or diabetes mellitus.

The study was approved by the ethics committee for the capital region of Denmark (journal number H-1-2011-033). The study was conducted according to the Declaration of Helsinki, and all the participants signed a written consent prior to the experiment.

### Hearing Aid Fitting and Signal Processing

Both HA fitting and signal processing used in the present study were identical to those in Alickovic et al. (2020). All participants were fitted with commercially available HAs (Oticon Opn S 1^TM^ mini-Receiver-in-the-ear) and power domes. The HAs were fitted using proprietary software based on each participant’s pure-tone hearing thresholds before the test session.

There were two conditions of signal processing. In the conditions where the NR scheme was OFF, the microphone was set to the omni-directional mode with an added natural slight forward effect in order to simulate the acoustic effect of the pinna. Amplification was provided using the Voice Aligned Compression (VAC+) rationale, which is a quasi-linear fitting rationale based on the loudness data from Buus and Florentine (2001). The VAC+ rationale has low compression knee points (between 20 and 50 dB SPL) in order to provide more compression at low input levels and less compression at high input levels. In the conditions when NR scheme was ON, a fast-acting version of a minimum-variance distortionless response beam-former was applied, which uses spatial filtering in order to attenuate background noise coming from behind the listener (Kjems and Jensen, 2012). Additionally, a single-channel postfilter was applied to further attenuate background noise (Jensen and Pedersen, 2015). Wendt et al. (2017) and Ohlenforst et al. (2018) have likewise used these settings.

In order to verify the technical effect of the NR scheme in creating contrast between the two foreground talkers and the four background babble signals, output signal-to-noise ratio (SNR) measurements were performed in the experimental setup using the Hagerman and Olofsson (2004) phase-inversion technique. A pair of HAs were fitted to the ears of a head-and-torso simulator (HATS) using closed ear tips. The experimental stimuli (see section “Stimuli”) were played back in the same way as in the EEG experiment and recorded *via* the HATS internal microphones. A second set of recordings was obtained with the phase of the background babble inverted, such that the foreground speech and the background babble could be separated from the mixture at the output of the HAs (Hagerman and Olofsson, 2004). The obtained articulation-index weighted output SNR using this method was 6.6 dB with NR OFF and 12.9 dB with NR ON, yielding a 6.3-dB SNR improvement provided by the tested NR scheme.

### Paradigm

#### Experimental Design

**FIGURE 2:**
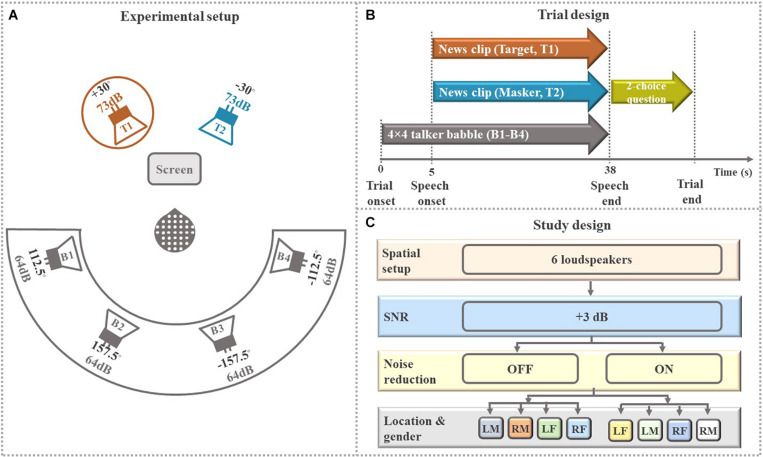
Overall study design. **(A)** Schematic illustrations of the experimental setup. EEG data were recorded from participants who were asked to selectively attend to one of two simultaneous speech stimuli (talkers T1, T2) coming from two loudspeakers positioned ± 30° to the left or right of the center. Speech stimuli were presented at an average intensity of 73 dB SPL each in the presence of 16-talker (4 × 4 talker) babble noise (B1-B4) coming from four rear loudspeakers positioned ± 112.5° and ± 157.5° to the left or right at an average intensity of 64 dB SPL each. Stimuli T1 and T2 were news clips (continuous speech) uttered by a male and a female talker. The circle around talker T1 shows that the participant in this trial was instructed to attend to the talker T1 and ignore the contralateral talker T2 and 16-talker babble noise (B1-B4). The participants were comfortably seated and instructed to fixate their eye-gaze at the computer screen during sound presentation. **(B)** Schematic illustration of the trial sequences. **(C)** Schematic illustration of the study design. The two conditions “+3 dB OFF” and “+3 dB ON” consisted of 20 trials each, divided into four sub-block of five randomized consecutive trials with target talker being “left mail (LM),” “right male (RM),” “left female (LF),” and “right female (RF).” The presentation order of these four subconditions was counterbalanced across participants for each condition.

The experimental design in the present study was inspired by prior work with NH listeners (Das et al., 2018) and HI listeners (Alickovic et al., 2020). The experiment took place in a double-walled sound-proof booth with controlled light conditions. As shown in Figure 2A, the participants sat comfortably in the center of a circle of six loudspeakers positioned at ±30°, ±112.5° and ±157.5° azimuth relative to the participants. At the two loudspeakers (*T*_1_ and *T*_2_) in the foreground, the attended talker (target) and contralateral ignored talker (masker) were presented, symmetrically off-center to counterbalance any asymmetrical hearing abilities. The four loudspeakers in the background (*B*_1_-*B*_4_), each presenting 4-talker babble noise, were to increase task complexity. The instructions and the questions appeared on the computer screen in front of the participants placed in a way to avoid acoustic shadowing.

#### Stimuli

All speech streams consisted of Danish news clips of neutral content to avoid emotional responses in the EEG traces. The talkers were selected because they had approximately the same word pace and intonation. To eliminate gaps in the audio streams, all silences lasting longer than 200 ms were shortened to 200 ms.

Figure 2 shows how the foreground talkers and background babble noise were presented from six loudspeakers. The two speech streams in the frontal hemifield (foreground) were read by the same male and female talker for all trials. Each of the foreground streams were presented from a single loudspeaker. Each of the four loudspeakers in the rear hemifield (background) played a four-talker babble comprising two female speakers and two male speakers, with none of the 16 streams being equal for each trial. Stimulus amplitudes in of the male and female talker in the foreground were normalized to the same overall root mean squared (RMS) intensity. The long-term average spectrum of the babble noise was matched to the overall spectrum of male and female talkers in the foreground to ensure homogeneous masking. Speech stimuli in the foreground were presented at 73 dB SPL for each loudspeaker, while the babble noise from each of the four background loudspeakers was presented at 64 dB SPL, leading to a total background level of 70 dB SPL.

Stimuli were routed through a sound card (RME Hammerfall DSP multiface II, Audio AG, Haimhausen, Germany) and were played *via* six loudspeakers Genelec 8040A (Genelec Oy, Iisalmi, Finland).

#### Procedure

Participants listened to 80 trials presented in four blocks (four experimental conditions—four different HA settings) with each block lasting for about 20 min. Similar to our previous study (Alickovic et al., 2020), the experiment in this study was arranged in the form of a randomized block design. Prior to testing, a training block of four trials was presented to familiarize the participants with the selective attention task. The participants were not given feedback at any time during the testing. As shown in Figure 2B, participants first heard the background noise in each trial. Five seconds later, the target and masker stimuli were presented simultaneously. After the stimuli were over, a two-choice question about the content of the attended speech was presented on the screen to ensure sustained attention.

As shown in Figure 2C, for this study, we used the data from two of the blocks (“NR OFF” and “NR ON”) in which NR schemes (active/ON and inactive/OFF) were tested. In one block (“NR OFF”), the NR feature was switched off, and in the other block (“NR ON”), the NR feature was switched on. The two remaining blocks were tested using another type of experimental HA signal processing and the data related to these two blocks are not reported in this manuscript. In total, we analyzed data from 40 trials (2 blocks × 20 trials). In each trial, the target and masker speech (T1 and T2) in the frontal hemifield were spoken by a male and a female talker. The participants were asked to selectively attend to one of the two talkers in the frontal hemifield (target talker), while ignoring irrelevant parts of the scene comprising the opposite-sex masker talker from contralateral location in the frontal hemifield and the background babble noise in the rear hemifield. Each block was further divided into four sub-blocks of five randomized consecutive trials for each of “left male (LM),” “right male (RM),” “left female (LF),” and “right female (RF).” Before each sub-block, a visual cue was displayed on the screen indicating talker (male or female) and the side to be attended. In addition, 5 s of the to-be-attended speech was presented simultaneously from the to-be-attended loudspeaker location, allowing the participants to prepare for the auditory task. The participants were given a rest period between blocks.

Two pairs of identical HAs were used for the two blocks presented in this study. In one pair of HA, the NR scheme was turned off, whereas the other pair of HAs had the NR scheme activated. The HAs were removed and replaced between the blocks. The NR scheme was active (ON) and one trial for each of the stimuli “LM,” “RM,” “LF,” and “RF” was presented in the training block to ensure that the participants could perform the task.

### Neural Data Acquisition

Electroencephalography data were acquired at a sampling rate (*f*_*s*_)of 1,024Hz with a BioSemi ActiveTwo recording system (Amsterdam, Netherlands). Recordings were done with 64 active Ag/AgCl electrodes. Two additional electrodes (an active electrode, CMS, common mode sense, and a passive electrode, DRL, driven right leg) served as the reference for all other recording electrodes and two additional electrodes were placed on the mastoids. Electrodes were mounted over the scalp according to the International 10–20 system. To maintain stable and high-quality electrical connections between electrodes and scalp, the electrodes were prepared (and if necessary, supplied with additional gel) such that the electrode offset voltages were stable and limited (the absolute voltages were below 50 mV).

### Components of EEG-Based Stimulus Reconstruction

For preprocessing and data analysis, we used the Fieldtrip toolbox [version 20181231; Oostenveld et al. (2011)], mTRF Toolbox [version 1.3, Crosse et al. (2016)], and custom-written scripts in MATLAB (R2018a, MathWorks).

#### Neural Data Preprocessing

Electroencephalography signals were first epoched from −15 to 48 s, relative to the onset of the target and masker stimuli (T_1_–T_2_). Ten seconds of EEG data before and after any stimulus (*T*_1_, *T*_2_, *B*_1_, *B*_2_, *B*_3_, and *B*_4_) were used as buffer zones for filtering edge artifact (van Driel et al., 2020). The raw EEG data was referenced to the average of the two mastoid channels. The broadband EEG was then digitally bandpass filtered between 0.5 and 70 Hz (a zero-phase Hamming window FIR, filter order: 3*f*_*s*_/*f*_*c*_ with *f_c_* being the lower cutoff frequencies, *fir1* function in Matlab), while an additional narrow-band notch filter (49–51 Hz, zero-phase Hamming window FIR, filter order: 3*f*_*s*_/*f*_*c*_) was set to remove remaining line noise. The signals were filtered both forward and backward using the *filtfilt* function in MATLAB in order to eliminate any phase shifts or delays in the filtered signals. We then downsampled to 256 Hz to decrease subsequent processing time. The EEG channels contaminated by noise were visually inspected and removed. We rejected an average of 2.2 (SD = 2.3) channels. In place of the bad channels, data were interpolated from the surrounding clean EEG channels using the nearest neighbor method in Fieldtrip (Oostenveld et al., 2011). We then applied denoising using independent component analysis [ICA, Bell and Sejnowski (1995); Delorme and Makeig (2004)] and manually rejected components that were clearly related to the residual artifacts caused by eye movements, eye blinks, muscle activity, heart beats, and single-channel noise. On average 14.6 (SD = 4.6) components were removed. One participant with excessively noisy EEG data was excluded from further EEG analysis. Moreover, one block of data of one participant was also excluded from the further EEG analysis due to technical problems. Finally, EEG signals were bandpass filtered between 2 and 8 Hz (neural δ and θ bands) using a third-order Butterworth filter, downsampled to 128 Hz and segmented into trials of 33-s duration from 0 to 33 s relative to the onset of the target stimuli.

#### Stimulus Envelope Extraction

To later estimate their neural representation in the auditory scene, we extracted the envelopes of each element in the auditory scene. Similar to our previous study (Alickovic et al., 2020), the envelope *U_X_* was extracted by taking the absolute values of the analytic signal of the original sound stream *X* (*hilbert* function in MATLAB), low-pass filtering it with a cutoff at 8 Hz using a third-order Butterworth filter (O’Sullivan et al., 2015), and then resampling the resulting waveforms to 128 Hz and separating them into 33-s snippets to match their corresponding EEG trials.

For the present study, we extracted five different envelopes of five elements in the auditory scene (i.e., target, masker, foreground, background noise, and entire acoustic scene, see Figure 2A):

(1) Target envelope *U_T_*: the envelope of the target speech stream *T*.
(2) Masker envelope *U_M_*: the envelope of the masker speech stream *M*.
(3) Foreground envelope *U_F_*: the envelope of the foreground stream *F*, comprising both target and masker speech streams *T*, *M*, and expressed as sum of their waveform *F* = *T* + *M*.
(4) Background noise envelope *U_B_*: the envelope of the background noise stream *B*, comprising four different four-talker babbles in the rear hemifield *B*_1_, *B*_2_, *B*_3_, *B*_4_, and expressed as sum of their waveform *B* = *B*_1_ + *B*_2_ + *B*_3_ + *B*_4_.
(5) Acoustic scene envelope *U*_*ALL*_: the envelope of the entire acoustic scene, comprising all elements in the scene (i.e., target, masker and background babble noise *T*, *M*, *B*_1_, *B*_2_, *B*_3_, and *B*_4_), and expressed as sum of their waveform *ALL* = *T* + *M* + *B*_1_ + *B*_2_ + *B*_3_ + *B*_4_. Stimulation level was not considered.

For decoders with multiple speakers (i.e., foreground, background noise and entire acoustic scene), we computed the envelope of the sum of the audio (speech stimuli).

The extracted envelopes and EEG data are then used to estimate the (data-driven) neural models (decoders), as the next sections illustrate.

#### Stimulus Envelope Reconstruction Accuracy

Our analyses for the two testing conditions (inactive or active NR) were based on stimulus envelope reconstruction (here referred to as SR) method, i.e., estimating the backward modeling of multivariate EEG responses onto the stimulus envelope, which allowed assessing the extent of cortical representations of the five stimuli in the two conditions [see O’Sullivan et al. (2015), Alickovic et al. (2019), and Geirnaert et al. (2020) for a more detailed discussion on the method]. In order to assess how strongly each stimulus was represented in the evoked neural responses, we reconstructed an estimate of that stimulus envelope from the EEG data using the mTRF Toolbox (Crosse et al., 2016). Envelope reconstruction accuracy–the Pearson’s correlation (*r*-value) between the reconstructed stimulus envelope and the actual stimulus envelope–served as a metric of the fidelity of the cortical representation of that stimulus envelope (Ding and Simon, 2012a; Presacco et al., 2016; Alickovic et al., 2020).

**FIGURE 3:**
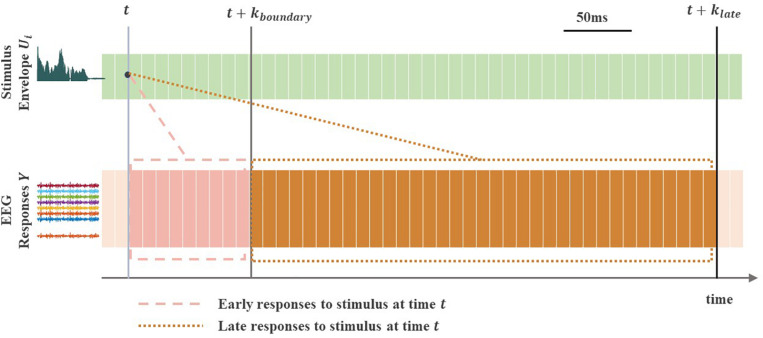
Early vs. late EEG neural responses to a continuous sound stimulus. The light gray vertical line indicates timepoint at arbitrary time ***t***. The gray and dark gray vertical lines indicate timepoints at times ***t* + *k*_{*boundary*}_** and ***t* + *k*_{*late*}_**, with ***k*_*bounary*_** and ***k*_*late*_** denoting the end points (time lags) of the two different (early and late) time integration windows. The early EEG responses to stimulus at time point ***t*** are shown in the dashed box, whereas the late responses are shown in the dotted box. The stimulus envelope ***U*_*i*_** can be reconstructed using EEG responses ***Y*** from early [in the **(*t*, *t* + *k*_{*boundary*}_)** range], late [in the **(*t* + *k*_{*boundary*}_, *t* + *k*_{*late*}_)** range] or overall [in the **(*t*, *t* + *k*_{*late*}_)** range] integration window, which allows a direct comparison between the three separate reconstructions (***r***-values).

To reconstruct the envelope from the corresponding EEG responses, a linear decoding model was formulated as ${\hat{U}}_{i}\in \left\{T,M,F,B,ALL\right\}\left(t\right)=\sum_{j=1}^{n_{y}}\sum_{k=k_{b}}^{k_{e}}Y_{j}\left(tk\right)A_{ij}\left(k\right)$, where ${\hat{U}}_{i}\left(t\right)$ is the reconstructed stimulus envelope at time *t*, *Y*_*j*_(*t*) is filtered EEG signal from channel *j*, *n_y_* is the number of EEG channels, *A*_*ij*_(*k*) is a linear decoder for channel *j* and *k* is an appropriately chosen time integration window (from the beginning time *k_b_* to end time *k_e_*) over which the decoder *A*_*ij*_ is estimated. Choosing appropriate values of *k_b_* and *k_e_* is important when comparing the reconstruction accuracy (*r*-values) from any desired time integration window. The decoder was estimated using dense (*l*_2_-regularized) linear regression (Alickovic et al., 2016, 2019). Figure 3 illustrates the difference between reconstructing of the stimulus envelope from EEG response using either early or late time integration window. The cutoff time point between early and late EEG responses to sound stimulus, *k_boundary_*, was the single value of 85 ms (i.e., *k*_boundary_ = 85ms) for all subjects [this time parameter was taken from MEG data reported in Puvvada and Simon (2017)]. When reconstructing the stimulus envelope from early EEG responses only, the time integration window ranged from 0 to 85 ms (*k*_*b*_ = 0 to *k*_*e*_ = *k*_*boundary*_), which we refer to as the early integration window. When reconstructing the stimulus envelope from late EEG responses only, the time integration window ranged from 85 to 500 ms (i.e., *k*_*b*_ = *k*_boundary_ to *k*_*e*_ = *k*_late_ where *k*_late_ = 500ms, see Figure 3), which we refer to as the late integration window.

#### Classification Accuracy

As a measure for how selectively the target talker is cortically represented in contrast to the masker talker, classification accuracy was assessed. First, two Pearson’s *r*−*values*were calculated: the reconstructed envelope of the target talker (using the target decoder) was both correlated with (1) the actual target envelope and (2) with the actual masker stimulus envelope. Since the decoder was trained on the target talker and given that there is a difference between the representation of the target and the masker talker, the envelope of the target talker should reach higher correlations than the envelope of the masker talker. Classification accuracy refers to the percentage of trials in which this was the case. Classification accuracy can be interpreted as a measure of cortical selectivity

#### Time Window Analysis

It is worth noting that the overall time-windows covering all time lags between 0 and *k*_*late*_ following the speech onset are commonly used in the literature that investigates the relationship between cortical responses and speech. We therefore investigated if using the overall integration window covering overall (i.e., both early and late) EEG responses ranging from 0 to 500 ms (*k*_*b*_ = 0, *k*_*e*_ = *k*_late_ where *k*_late_ = 500ms, see Figure 3) further improved envelope reconstructions compared to using late responses only.

Because the goal of the study was to assess the effect of NR on cortical processing of speech, we further investigated whether activating the NR scheme reduced the size of the late and overall integration windows required to maximize the stimulus reconstructions. Here, similar to Presacco et al. (2016, 2019), four different values were used for the end point *k*_*late*_: 150, 250,350, *and* 500ms. One major reason for narrowing the end points of late and overall integration windows down to 150 ms is that several MEG studies have shown that cortical speech representation substantially worsens as the window decreases down to 100 ms.

#### Decoder Design

To assess quantitatively whether NR affected the representations of different stimuli (see section “Stimulus Envelope Extraction”) in early and/or late neural (EEG) responses and across different time windows, we estimated five different potential cortical representations of five elements in the multi-talker auditory scene. For each envelope type (i.e., target and masker speech, foreground, background noise, and entire acoustic scene), a separate decoder was built:

(1) Target decoder (*A_T_*): the decoder trained on the responses to the target speech.
(2) Masker decoder (*A_M_*): the decoder trained on the responses to the masker speech.
(3) Foreground decoder (*A*_*F*_): the decoder trained on the responses to the foreground speech.
(4) Background noise decoder (*A*_*B*_): the decoder trained on the responses to the background noise.
(5) Entire acoustic scene decoder (*A*_*ALL*_): the decoder trained on the responses to the entire scene.

All five decoders were built for early, late, and overall EEG responses separately.

Decoders were estimated for each individual participant and for each of the experimental conditions separately in a similar way to our previous study (Alickovic et al., 2020). Each specific decoder was then used to reconstruct the corresponding envelope. The decoders were trained and tested separately for each experimental condition and for each time integration window using a leave-one-out (LOO) cross-validation (CV) scheme. A decoder, trained on all but one trial in a per-trial manner [Equation 25, Alickovic et al. (2019)], was used to reconstruct the envelope of the left-out trial and to find the envelope reconstruction accuracy by calculating the linear (Pearson’s *r*) correlation between the reconstructed envelope and the actual envelope [see O’Sullivan et al. (2015) and Alickovic et al. (2019) for more details on the “per-trial” training approach]. The CV procedure was then repeated for each of the 20 trials per experimental condition (Crosse et al., 2016). To avoid over-fitting, a range of decoders were constructed using different values of (ridge) regularization parameter λ between 10^−1^ and 10^7^ [Equations 19 and 20, Alickovic et al. (2019)]. The λ value that produced the highest envelope reconstruction accuracy (i.e., the highest group-mean LOO Pearson’s *r* correlation), averaged across trials, experimental conditions and participants, was selected.

The foreground decoder and entire acoustic scene decoder (built using early EEG responses) test the hypothesis (H1) that the cortical representations of the foreground and entire acoustic scene will be more enhanced in early responses with the NR ON compared to OFF. The target decoder analysis tests the hypothesis (H2) that the target talker is selectively represented in late responses. The target decoder, masker decoder and background noise decoder (built using late EEG responses) test the hypothesis (H3) that the active NR scheme enhances the cortical representations of the target and masker talkers in the foreground and suppresses the noise in the background in the late responses. Finally, the analysis of different post-stimulus integration windows (early, late and overall) used to build these five decoders includes an additional exploration of the EEG responses to investigate whether the active NR scheme reduces cortical processing time required to maximize reconstructions of the stimuli.

### Statistical Tests

#### Reconstruction Accuracy

All statistical analyses were performed using R (version 3.6.2) software. First, the normality and homoscedasticity assumptions were checked to ensure a good fit. Next, several linear mixed models (LMMs) were estimated using the lme4 (Bates et al., 2014) and lmerTest (Kuznetsova et al., 2017) packages. All LMMs in this study were fitted using the REML criterion. The significance criterion of α=0.05 was adopted. Significance levels for fixed LMM factors were computed using Satterthwaite approximation for degrees of freedom [*anova* function in the lmerTest package, which gives a type III analysis of variance (ANOVA)]. Significant effects for the fixed factors from the LMM ANOVAs were followed by post-hoc pairwise analyses using differences of least-squares means (*difflsmeans* function in the lmerTest package), and adjusting the *p*-values for multiple comparisons using the false discovery rate (FDR) method (*p.adjust* function in the stats package, *BY* option). Participants and trials were treated as random factors for all LMM ANOVAs (Barr et al., 2013).

To test hypotheses H1-H3, we first applied one-way LMM ANOVA to investigate the effect of NR (NR, two types: ON vs. OFF). We fitted one LMM ANOVA model for each of the five decoder types and each of the time windows:

(1) Early in the [0, 85 ms] range used to answer H1,
(2) Late [85–500 ms] range used to answer H2 and H3, and
(3) Overall [0–500 ms] range used to conduct an additional analysis, testing for the effect of different time windows on cortical representation.

Here, we modeled reconstruction accuracy as a function of NR type (fixed factor).

We then performed two-way LMM ANOVAs to investigate effects of NR, effects of end times of different integration windows (end integration window *k*_late_, four levels: 150, 250, 350, and 500 ms), NR × end time interactions on late and overall EEG responses separately. Fixed factors in the LMM ANOVAs included the NR type and end integration window, and random factors were as before. We fitted one LMM model for each of the decoders estimated using either late or overall integration window. NR × end integration window interactions were assessed to determine if increasing levels (lengths) of end integration window affect cortical representation of different stimuli during OFF and ON NR schemes differently.

Next, we performed two-way LMM ANOVAs to investigate effects of NR [NR, two types: inactive (OFF) vs. active (ON)], effects of time integration windows (integration window, two ranges: late vs. overall), NR × window interactions. The NR type and integration window were treated as fixed factors. Participants and trials were included as random factors. We fitted one LMM model for each of the decoders.

Furthermore, we performed two three-way LMM ANOVAs to investigate effect of NR (two levels: OFF, ON), effect of EEG responses windows (2 levels: early, late), effect of individual elements (five levels: target, masker, foreground, background noise, and acoustic scene envelopes), and their interactions on reconstruction accuracy. Fixed factors in the LMM ANOVAs included the NR type, EEG response window and individual element. Finally, we performed a three-way LMM ANOVA to investigate effect of NR (OFF vs. ON), effect of EEG responses windows (early vs. late), effect of attention (target vs. masker), and their interactions on reconstruction accuracy. Fixed factors in the LMM ANOVAs included the NR type, EEG response window and attention. Random factors were in all three-way ANOVAs were as before.

#### Classification Accuracy

To asses classification performances, see section “Classification Accuracy,” we determined that, at group level, a score of at least 65.625% was significantly higher than the theoretical chance level of 50% (*p* = 0.05) based on a binomial distribution using 32 participants per NR scheme condition. Similar to Combrisson and Jerbi (2015), we used the MATLAB function *binoinv* to compute the statistically significant threshold (empirical chance level) *St*(*p*) = *binoinv*(1 − *p*, *n*, 1/*c*) × 100/*n*, where p is the significance level, n is a sample size and *c* is a number of classes.

#### Behavioral Performances

To assess behavioral performances, the percentage of correct responses for the two-choice questions was obtained for each of the two NR scheme conditions. A one-way repeated-measures (RM) ANOVA was performed to investigate the effect of the NR schemes on behavioral performance.

## Results

We presented two simultaneous talkers in the frontal hemifield (foreground) and babble noise in the background. Participants were asked to attend one of the talkers in the foreground (target) and to ignore the other (masker). We examined the effect of NR processing on the cortical representation of the target talker, masker talker, foreground, background noise and entire acoustic scene across different time integration windows. The strength of cortical representation was assessed by the reconstruction accuracy, which is the Pearson’s correlation between the reconstructed audio envelopes and the actual audio envelopes.

### Early Neural Responses

#### Noise Reduction Effects for the Foreground and Entire Acoustic Scene Mixture

**FIGURE 4:**
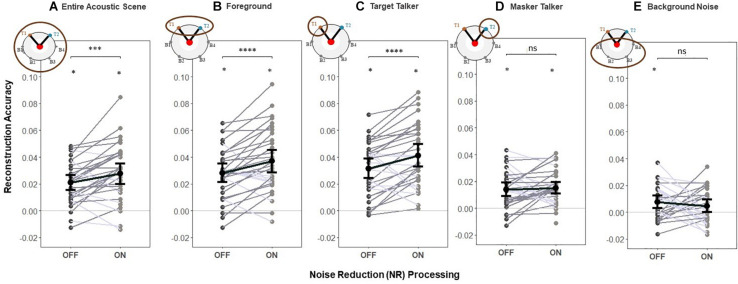
Reconstruction accuracy for early responses (0–85 ms). (H1) The NR scheme enhances the representation of the entire acoustic scene, foreground and target in early EEG responses. A significant main effect of NR scheme setting was observed, indicating the enhanced neural representations of the entire acoustic scene, foreground and target envelopes in early EEG responses when NR scheme was active. For the masker talker and background noise, results indicate no significant difference in reconstruction accuracy between NR scheme settings. Shaded dots indicate trial-averaged individual reconstruction accuracy results (individual level), whereas black dots and error bars show the group (grand-average ± 1 between subject SEM) level reconstruction accuracy results using decoders trained only on **(A)** entire acoustic scene envelope (entire acoustic scene decoder), **(B)** foreground envelope (foreground decoder), **(C)** target envelope (target decoder), **(D)** masker envelope (masker decoder), and **(E)** background noise envelope (background noise decoder). Each shaded dot represents the reconstruction accuracy of the envelopes for a single participant (i.e., the reconstruction accuracy values averaged over 20 trials), each line in gray denotes single participant (dark gray denotes enhanced reconstruction accuracy and light gray denotes reduced reconstruction accuracy when activating NR scheme) and each line in black denotes average reconstruction accuracy across participants for each envelope type. The asterisk above each condition indicates reconstruction performance significantly greater than zero (determined with a two-sided *t*-test with criterion *p* = 0.05). The asterisk above the bars indicates significant differences between the NR conditions (*p = 0.05, **p = 0.01, ***p = 0.005, and ****p = 0.0001).

To test H1 (that NR would increase reconstruction accuracy for the foreground sounds and the entire acoustic scene), we used the foreground and entire acoustic scene decoders to reconstruct the foreground and acoustic scene envelopes, respectively. We contrasted the envelope reconstruction accuracy for the entire acoustic scene and foreground envelopes in early EEG responses when the NR scheme was active compared to that when it was inactive. Group (i.e., averaged over participants) and individual (i.e., single participant) reconstruction accuracy are shown in Figures 4A,B, respectively.

One-way LMM ANOVAs revealed significant main effects of NR for both the entire acoustic scene (*F*_1,1273_ = 10.6, *p* = 0.0012, see Figure 4A) and foreground (*F*_1,1272_ = 16.6, *p* = 5.01*e*−5, see Figure 4B). At the individual level, 22/32 and 25/32 individuals had higher reconstruction accuracy for the entire acoustic scene and foreground envelopes, respectively, when the NR scheme was turned on compared to when it was turned off. This indicates that the early EEG responses represented the entire acoustic scene and foreground with significantly higher fidelity when the NR scheme was turned on compared to when it was turned off.

#### Noise Reduction Effects for the Target, Masker and Background Noise

We also conducted an additional exploration of how NR affected the reconstruction accuracy of single parts of the acoustic scene by breaking down the analysis into separate decoders for the target, masker, and background (Figures 4C–E). One-way LMM ANOVA showed a significant effect of NR on reconstruction accuracy of the target (*F*_1,1272_ = 18.3, *p* = 2.1*e*−5, see Figure 4C). Results indicate no significant difference in reconstruction accuracy between NR scheme settings for the masker (*F*_1,1278_ = 1.3, *p* = 0.2517, see Figure 4D) nor for background (*F*_1,1262_ = 1.5, *p* = 0.2173, see Figure 4E).

This additional analysis suggests that the hypothesized effect of NR on reconstruction accuracy of the foreground and entire acoustic scene found above (shown in Figures 4A,B) may in fact be driven by NR-related improvements in reconstruction accuracy specifically for the target, even in the early responses.

Furthermore, target vs. masker classification analysis (see section “Classification Accuracy”) was done separately to determine how well the target source could be classified from early EEG responses. We did not expect a large effect of attention in early responses, which would result in target vs. masker classification accuracies not significantly greater than chance (chance level was 65.625% based on a binomial distribution using 32 participants per NR scheme condition, see section “Classification Accuracy”). The classification accuracy was below the chance level when the NR scheme was turned off (63.0%). However, classification accuracy was above the chance level (69.2% correct classification) and was significantly higher when NR was turned on compared to that when it was turned off (*F*_1,1280_ = 5.6, *p* = 0.0178; one-way LMM ANOVA). Taken together, classification analysis results suggest a significant effect of NR and a weak but significant effect of attention on early EEG responses when the NR was turned on.

### Late Neural Responses

#### Noise Reduction Effects for the Foreground and Entire Acoustic Scene Mixture

**FIGURE 5:**
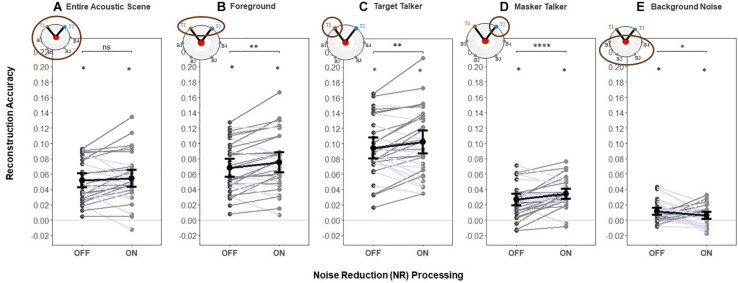
Reconstruction accuracy for late responses (85–500 ms). (H2-H3) The target talker is represented with significantly higher fidelity than masker talkers in late EEG responses. The NR scheme enhances the representation of the foreground and individual (target and masker) talkers in the front hemifield (i.e., higher reconstruction accuracy) and suppresses the representation of the background noise (i.e., reduced reconstruction accuracy) in late EEG responses. A significant main effect of attention, indicating selective cortical representations of the target envelope in the late EEG responses, was observed. A significant main effect of NR scheme setting on reconstruction accuracy was observed, indicating the enhanced cortical representations of the target, masker, and foreground envelopes and the reduced representation of the background noise envelope in the late EEG responses when NR scheme was active. Gray dots indicate trial-averaged individual reconstruction accuracy results, whereas black dots and error bars show the group (grand-average ± 1 between subject SEM) reconstruction accuracy results using decoders trained only on **(A)** entire acoustic scene envelope (entire acoustic scene decoder) or **(B)** foreground envelope (foreground decoder) or **(C)** target envelope (target decoder) or **(D)** masker envelope (masker decoder) or **(E)** background noise envelope (background noise decoder). Each horizontal line in gray denotes single participant (dark gray denotes enhanced cortical representation and light gray denotes reduced cortical representation when activating NR scheme) and each horizontal line in black denotes average reconstruction accuracy across participants for each envelope type. The asterisk above each condition indicates reconstruction performance significantly greater than zero (determined with a two-sided *t*-test with criterion *p* = 0.05). The asterisk above the bars indicates significant differences between the noise conditions (*p = 0.05, **p = 0.01, and ****p = 0.0001).

We reconstructed the envelopes of the entire acoustic scene and the foreground using separate foreground and entire acoustic scene decoders, respectively. Figures 5A,B shows the group (i.e., average over participants) and individual (i.e., single participant) reconstruction accuracy.

In the first analysis, we compared the effect of NR on the reconstruction accuracy for the foreground compared to the entire acoustic scene based on the late responses. One-way LMM ANOVA showed no significant difference between NR scheme settings for the entire acoustic scene (*F*_1,1251_ = 1.5, *p* = 0.2158, see Figure 5A). However, there was significantly higher reconstruction accuracy with NR on compared to off for the foreground (*F*_1,1250_ = 7.7, *p* = 0.0055, see Figure 5B).

#### Noise Reduction Effects for the Target, Masker and Background Noise

To evaluate the hypothesis H3 that the NR scheme enhances the representation of the target and masker talkers in the front hemifield and suppresses the representation of the background noise in the rear hemifield in late responses, we compared reconstruction accuracy with NR on vs. off for the specific parts of the acoustic scene. We reconstructed the envelopes of the target, masker and background noise using separate target, masker and background noise decoders, respectively. Figures 5C–E shows the group (i.e., average over participants) and individual (i.e., single participant) reconstruction accuracy.

One-way LMM ANOVAs showed significant effects of NR on reconstruction accuracy for the target (Figure 5C, *F*_1,1250_ = 6.9, *p* = 0.0084), as well as the masker (Figure 5D, *F*_1,1276_ = 11.2, *p* = 0.0009), indicating enhanced cortical representation of both the target as well as the masker when the NR scheme is turned on compared to off. At the individual level, 20/32 and 22/32 individuals showed higher reconstruction accuracy for the target and masker, respectively, when the NR scheme was turned on compared to when it was turned off.

Conversely, a one-way LMM ANOVA analysis of the background noise envelope reconstruction accuracy between two NR settings (on or off) showed that reconstruction accuracy for the background noise is significantly reduced when the NR scheme is turned on compared to when it is turned off (Figure 5E, *F*_1,1262_ = 6.2, *p* = 0.0124).

#### Attention Effects for the Target and the Masker in Late Responses

To test H2 (that the late EEG responses selectively represent the target talker with significantly higher fidelity than the masker talker), we created an additional one-way LMM ANOVA to compare reconstruction accuracy for the target with the masker in late EEG responses. In this model, reconstruction accuracy was treated as a dependent measure, the variable attention (attention, two levels: target vs. masker) was treated as a fixed effect, and the participants and trials were treated as random effects. We found a significant effect for attention (*F*_1,1942_ = 1130.8, *p* = 2.2e−16), with reconstruction accuracy higher for the target talker compared to the masker.

A similar pattern was observed when examining classification performances (i.e., how well the target vs. masker sources could be classified from late EEG responses, see section “Classification Accuracy”). We expected to see a large effect of attention in late responses, which would result in classification accuracies significantly greater than chance (see section “Classification Accuracy”). The grand average classification accuracies were significantly larger than chance when NR was on (85.8% correct classification) and off (87.4% correct classification). Together, these results confirm that the late EEG responses selectively represent the target talker with significantly higher fidelity than masker talker.

### Comparison of Early vs. Late Responses

**FIGURE 6:**
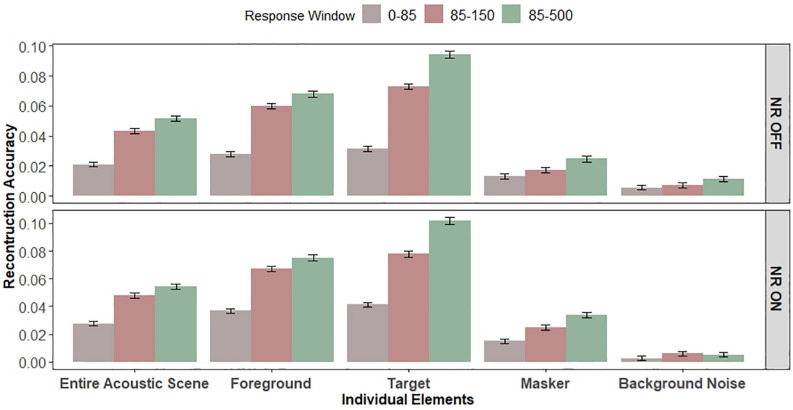
Early vs. late EEG responses. Bar graphs show the average reconstruction accuracy tested for the early (0–85 ms) and late (85–150 and 85–500 ms) integration windows. Left and right panel show the reconstruction accuracy for “NR OFF” and “NR ON” conditions, respectively. Error bars indicate SEM. Significant main effects of EEG response window, NR and individual elements (envelopes) and significant interaction between the response window and individual elements and between the NR and individual envelope were observed. Significant effect of attention (target vs. masker) on early and late EEG responses was observed.

Next, to contrast early (0–85 ms) vs. late (85–500 ms) responses, we created a separate one-way LMM ANOVA (collapsed across the two NR schemes and five different envelope types) to test the effect of the response window on the reconstruction accuracy. A one-way LMM ANOVA showed significant differences in reconstruction accuracy values between early (0–85 ms) and late (85–500 ms) integration windows (*F*_1,12340_ = 1144.3, *p* = 2.2*e*−16), indicating enhanced envelope processing in the late EEG responses. This can be seen by comparing the overall size of the reconstruction accuracy values in Figure 6.

Finally, we have done additional analysis comparing the reconstruction accuracy values for early vs. late EEG responses using approximately the same window sizes (0–85 ms and 85–150 ms for early and late integration windows, respectively). In the previous LMM ANOVAs, we compared the decoders with different number of parameters, as early and late integration windows were of different sizes (i.e., 85 and 415 ms window duration for early and late integration windows, respectively), and thus decoders of different power. If the observed significant effects were driven only by the number of parameters, and not by attention, it would be surprising to see the differences in reconstruction accuracy values between early and late integration windows of approximately same sizes. However, a one-way LMM ANOVA, collapsed across NR schemes and envelope types, showed significant improvements of the reconstruction accuracy values for the late (85–150 ms) integration window, as compared to early (0–85 ms) integration window (*F*_1,12966_ = 626.3, *p* = 2.2*e*−16), confirming enhanced envelope processing in the late EEG responses.

### Relationships Among Early vs. Late Response Window, Noise Reduction, and Envelope Type

We created two three-way LMM ANOVAs to statistically compare the effects of EEG response window (two levels: early and late), NR (two levels: OFF and ON) and individual envelope (five levels: target, masker, foreground, background noise and entire acoustic scene) on the reconstruction accuracy, one where the late window size was 415 ms (i.e., 85–500 ms) and one where the late window size was 65 ms (i.e., 85–150 ms). Figure 6 re-plots the reconstruction accuracy values allowing easy comparison of the early and late EEG windows, NR scheme, and individual envelopes. The two three-way LMM ANOVAs revealed significant main effects of EEG response window (*F*_1,12321_ = 1,506.6, *p* = 2.2e−16;*F*_1,12321_ = 786.7, *p* = 2.2e−16, for the 85–500 ms and 85–150 ms windows, respectively), which is in line with the findings of the one-way LMM ANOVA analysis, NR (*F*_1,12419_ = 36.0, *p* = 2.1e−09;*F*_1,12413_ = 48.3, *p* = 3.8e−12, for the 85–500 and 85–150 ms windows, respectively) and individual envelope (*F*_4,12321_ = 790.0, *p* = 2.2e−16;*F*_4,12321_ = 680.3, *p* = 2.2e−16, for the 85–500 and 85–150 ms windows, respectively). We found significant interactions between the response window and individual envelope (*F*_4,12321_ = 167.6, *p* = 2.2e−16;*F*_4,12321_ = 93.5, *p* = 2.2e−16, for the 85–500 and 85–150 ms windows, respectively) and between the NR and individual envelope (*F*_4,12321_ = 9.5, *p* = 1.2e−07;*F*_4,12321_ = 6.1, *p* =  6.5e−05, for the 85–500 and 85–150 ms windows, respectively). The interactions between the response window and individual envelope and between the NR and individual envelope suggest that the envelope (i.e., individual elements) dependency on the reconstruction accuracy differs when the envelope is reconstructed from the late responses from the when it is reconstructed from the early responses, and when the NR scheme is ON from when the NR scheme is OFF.

To quantify the attention effects in early (0–85 ms) vs. late (85–500 and 85–150 ms, collapsed) responses, we created an additional three-way LMM ANOVA to statistically compare the effects of NR (OFF vs. ON) and attention (target vs. masker) on early and late EEG responses. A three-way LMM ANOVA revealed significant main effects of EEG response window (*F*_1,7134_ = 793.6, *p* = 2.2e−16), NR (*F*_1,7198_ =  37.3, *p* =  1.1e−09) and attention (*F*_1,7134_ = 1480.0, *p* = 2.2e−16). We observed a significant interaction between the EEG response window and attention (*F*_1,7134_ = 324.0, *p* = 2.2e−16) and a significant three-way interaction among EEG response window, NR, and attention (*F*_1,7134_ = 4.5, *p* = 0.0346) on the reconstruction accuracy. The interactions between the EEG response window and attention suggests that the attention (target vs. masker) effect is more prominent in the late EEG response window when compared to the early EEG responses. We did not observe significant interactions between the attention and NR (*F*_1,7134_ = 1.6, *p* = 0.1995) suggesting that the NR scheme was equally effective at enhancing both talkers. Furthermore, we did not find a significant effect for interaction between the EEG response window and NR (*F*_1,7134_ = 0.4, *p* = 0.50832) suggesting that the NR was equally effective at enhancing reconstruction accuracy at both EEG response windows.

### Noise Reduction Effects for Different Late Integration Window Sizes

**FIGURE 7:**
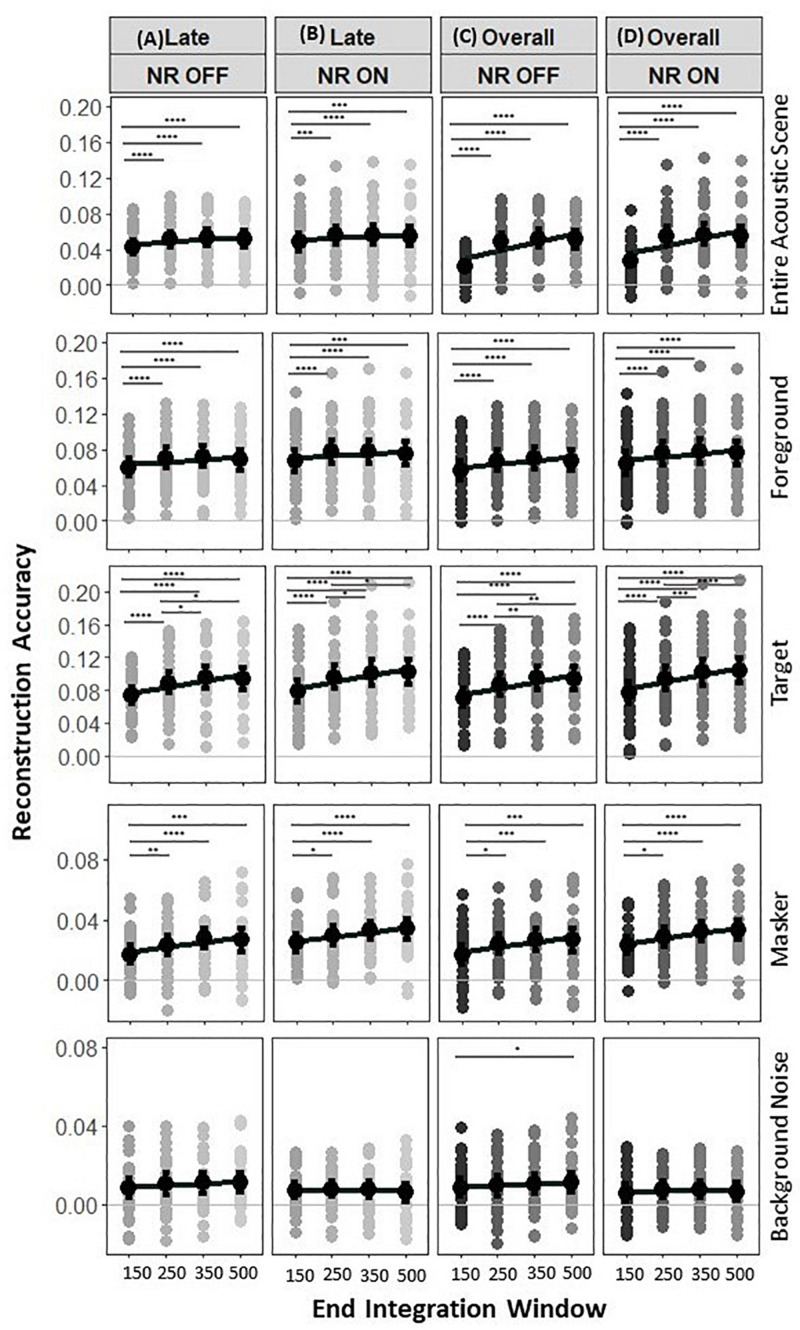
Reconstruction accuracy for late vs. overall EEG responses. Reconstruction accuracy in two experimental conditions (“NR OFF” and “NR ON”) tested for the four late integration windows tested for late responses only [left, **(A,B)**] and overall responses [(right, **(C,D)**]. No significant differences were found between reconstruction accuracy in the acoustic scene, foreground, target, masker and background noise envelopes from late and overall EEG responses (i.e., the envelopes of the acoustic scene, foreground, target, masker, and background noise can be reconstructed with similar accuracy from late EEG responses only compared to from the overall EEG responses). The NR and sizes of the late and overall integration windows have significant effects on reconstruction accuracy for the acoustic scene, foreground, target, and masker envelopes, with strong reconstruction accuracy values seen up to 350 ms for the target envelope and 250 ms for acoustic scene, foreground, and masker envelopes. The NR, but not the sizes of the late and overall integration windows showed significant effect on the reconstruction accuracy for the background noise. Each row of panels shows results for a different envelope type, i.e., entire acoustic scene envelope, foreground envelope, target envelope, masker envelope, and background noise envelope. Gray dots indicate trial-averaged individual reconstruction accuracy results, whereas black dots and error bars show the group (grand-average ± 1 between subject SEM) reconstruction accuracy results using decoders trained only on the relevant envelope. The asterisk indicates significant differences (*p = 0.05, **p = 0.01, ***p = 0.005, and ****p = 0.0001).

The previous analysis of the effect of NR on reconstruction accuracy in late responses used a fixed window size which we chose based on previous results from MEG studies [85–500 ms, (Puvvada and Simon, 2017)]. However, the analytic methods used to reconstruct the speech from the cortical responses perform differently depending on the duration of the analysis window. Therefore, we also evaluated the reconstruction accuracy for target, masker, foreground, background noise and acoustic scene envelopes using different integration window durations. The corresponding correlation values are shown in Figure 7, with a focus on the statistical measures pertinent to the integration window. A two-way LMM ANOVA, with main factors of NR and end times of late integration windows, was performed to analyze the effects of the integration window on the fidelity of the reconstruction of the target envelope.

Analysis of the entire acoustic scene envelope reconstruction accuracies again showed a significant effect of NR (Figures 7A,B, row 1, *F*_1,4531_ = 16.9, *p* = 3.0e−05), indicating again the enhanced neural representations of the acoustic scene envelope when the NR scheme is active compared to that when it is inactive, and significant main effect of end time of late integration window (*F*_3,4531_ = 18.5, *p* =  6.7e−12), indicating the increase in reconstruction accuracy with larger integration windows. No significant interaction was observed between the NR and end time of late integration window (*F*_3,4531_ = 0.4, *p* = 0.7294). The *post hoc* pairwise comparison of the acoustic scene reconstructed at 85–150 vs. 85–250, 85–150 vs. 85–350, and 85–150 vs. 85–500 ms at each NR scheme setting showed that the reconstruction accuracy of the acoustic scene envelope is significantly affected by the integration windows [NR OFF: *p* = 4.8e−05, *p* = 7.4e−08, *p* = 1.3e−05,NR ON: *p* = 7.5e−05, *p* = 2.6e−05, *p* = 0.0012for 85–150 vs. 85–250, 85–150 vs. 85–350, and 85–150 vs. 85–500 ms, respectively], whereas 85–250 vs. 85–350, 85–250 vs. 85–500, and 85–350 vs. 85–500 ms at each NR scheme setting showed that the reconstruction accuracy of the acoustic scene envelope did not have significantly lower values with end times of 350 and 500 ms than with and end time of 250 ms [NR OFF: *p* = 0.0.2805, *p* = 0.8126, *p* = 0.3997, NR ON: *p* = 0.8436, *p* = 0.5566, *p* = 0.4323 for 85–250 vs. 85–350, 85–250 vs. 85–500, and 85–350 vs. 85–500 ms, respectively]. Taken together, analysis of the acoustic scene envelope reconstruction accuracy showed that both the NR scheme and the end time of the late integration window have significant effects on the strengths of the cortical representation of the acoustic scene talker, with strong reconstruction accuracy seen with analysis window end times up to 250 ms.

Analysis of the foreground envelope reconstruction accuracies again showed a significant effect of NR (Figures 7A,B, row 2, *F*_1,4555_ = 43.4, *p* = 4.9e−11), indicating the enhanced neural representations of the foreground when the NR scheme is active compared to that when it is inactive, and a significant main effect of end time of late integration window (*F*_3,4534_ = 25.8, *p* =  2.2e−16), indicating the increase in reconstruction accuracy with larger integration windows. No significant interaction was observed between the NR and end time of late integration window (*F*_3,4534_ = 0.2, *p* = 0.8688). The *post hoc* pairwise comparison of the foreground envelope reconstructed at 85–150 vs. 85–250, 85–150 vs. 85–350, and 85–150 vs. 85–500 ms at each NR scheme setting showed that the reconstruction accuracy of the foreground was significantly affected by the integration windows [NR OFF: *p* = 2.7e−07, *p* = 2.1e−09, *p* = 6.3*e*−05,NR ON: *p* = 1.9e−07, *p* = 4.0e−07, *p* = 7.6*e*−05for 85–150 vs. 85–250, 85–150 vs. 85–350, and 85–150 vs. 85–500 ms, respectively], whereas 85–250 vs. 85–350, 85–250 vs. 85–500, and 85–350 vs. 85–500 ms at each NR scheme setting showed that the reconstruction accuracy of the foreground envelope did not have significantly lower values at 350 and 500 ms than at 250 ms [NR OFF: *p* = 0.3934, *p* = 0.2514, *p* = 0.0554, NR ON: *p* = 0.8891, *p* = 0.2103, *p* = 0.2655 for 85–250 vs. 85–350, 85–250 vs. 85–500, and 85–350 vs. 85–500 ms, respectively]. Taken together, analysis of the foreground envelope reconstruction accuracy showed that both the NR scheme and the end time of the late integration window have significant effects on the strengths of the cortical representation of the foreground envelope, with strong reconstruction accuracy seen with analysis window end times up to 250 ms.

Analysis of the target envelope reconstruction accuracies again showed a significant effect of NR (Figures 7A,B, row 3, *F*_1,4553_ = 28.4, *p* = 1.0e−07), indicating the enhanced neural representations of the target envelope when the NR scheme is active compared to that when it is inactive, and significant main effect of end time of late integration window (*F*_3,4533_ = 98.5, *p* =  2.2e−16), indicating the increase in reconstruction accuracy with larger integration windows. No significant interaction was observed between the NR and end time of late integration window (*F*_3,4553_ = 0.3, *p* = 0.8277). The *post hoc* pairwise comparison of the target reconstructed at 85–150 vs. 85–250, 85–150 vs. 85–350, 85–150 vs. 85–500, and 85–250 vs. 85–350 ms at each NR scheme setting showed that the reconstruction accuracy of the target talker was significantly affected by the integration windows [NR OFF: *p* = 2.2*e*−13, *p* =  2.2e−16, *p* = 2.2e−16, *p* = 0.004, *p* = 0.0147, NR ON: *p* = 4.3e−16, *p* = 2.2e−16, *p* = 2.2*e*−16, *p* = 0.01, *p* = 0.002 for 85–150 vs. 85–250, 85–150 vs. 85–350, 85–150 vs. 85–500 ms, 85–250 vs. 85–350 ms, and 85–250 vs. 85–500 ms, respectively], whereas 85–350 vs. 85–500 ms at each NR scheme setting showed that the reconstruction accuracy of the target envelope did not have significantly lower values at 500 ms than at 350 ms [NR OFF: *p* = 0.6825, NR ON: *p* = 0.6098]. Together, analysis of the target envelope reconstruction accuracy showed that both the NR scheme and the end time of the late integration window have significant effects on the strengths of the cortical representation of the target envelope, with strong reconstruction accuracy seen with analysis window end times up to 350 ms.

Analysis of the masker envelope reconstruction accuracies again showed a significant effect of NR (Figures 7A,B, row 4, *F*_1,4568_ = 51.6, *p* = 8e−13), indicating the enhanced neural representations of the masker envelope when the NR scheme is active compared to that when it is inactive, and significant main effect of end time of late integration window (*F*_3,4532_ = 14.9, *p* =  1.2e−09), indicating the increase of reconstruction accuracy with larger integration windows. No significant interaction was observed between the NR and end time of late integration window (*F*_3,4532_ = 0.4, *p* = 0.7641). The *post hoc* pairwise comparison of the masker talker reconstructed at 85–150 vs. 85–250, 85–150 vs. 85–350, and 85–150 vs. 85–500 ms setting showed that the reconstruction accuracy of the masker was significantly affected by the integration windows at each NR scheme [NR OFF: *p* = 0.0012, *p* = 1.6*e*−05, *p* = 0.0002, NR ON: *p* = 0.0235, *p* = 7.7e−05, *p* = 1.4e−05 for 85–150 vs. 85–250, 85–150 vs. 85–350, and 85–150 vs. 85–500, respectively], whereas 85–250 vs. 85–500 and 85–350 vs. 85–500 ms at each NR scheme setting showed that the reconstruction accuracy of the masker envelope did not have significantly higher values at 500 ms than at 350 or 250 ms [NR OFF: *p* = 0.5569, *p* = 0.5454, NR ON: *p* = 0.6953, *p* = 0.0820 for 500 vs. 350 ms and 500 vs. 250 ms, respectively]. Taken together, analysis of the masker envelope reconstruction accuracy showed that both the NR scheme and the end time of the late integration window have significant effects on the strengths of the cortical representation of the masker envelope, with strong reconstruction accuracy seen with analysis window end times up to 250 ms.

Analysis of the background noise envelope reconstruction accuracy values again showed a significant effect of NR (Figures 7A,B, row 5, *F*_1,4572_ = 13.7, *p* = 0.0002), indicating the reduced neural representations of the background noise envelope when the NR scheme is active compared to that when it is inactive. We did not observe significant effects of end time of late integration window (*F*_3,4534_ = 1.0, *p* = 0.3763), indicating that the reconstruction accuracy does not decline with smaller integration windows. No significant interaction was observed between the NR and end time of late integration window (*F*_3,4534_ = 1.3, *p* = 0.2626). Taken together, analysis of the background noise envelope reconstruction accuracy showed that the NR scheme but not the end time of the late integration window have significant effects on the strengths of the cortical representation of the background noise.

### Relationships Among Late Responses, Overall Responses, and Noise Reduction

Finally, we conducted an exploratory investigation of the neural representation of the target, masker, foreground, background noise and acoustic scene envelopes in overall EEG responses (using a time integration window covering both early and late EEG responses) and relationships among late responses, overall responses and NR. This investigation was done in order to determine whether the late responses and overall EEG responses show similar results each reconstructed type of envelope. These results are shown in Figures 7C,D.

We first examined the relationship between overall responses and NR. Similar to what has been previously reported for the analysis of the late responses, two-way LMM ANOVAs for the four overall integration windows (0–150, 0–250, 0–350, and 0–500 ms) fitted for target, masker, foreground, background noise and entire acoustic scene decoders separately showed a significant effect of NR on reconstruction accuracy for the target, masker, foreground, background noise and acoustic scene envelopes (Figures 7C,D, *F*_1,4553_ = 40.0, *p* = 2.8e−10, *F*_1,4564_ = 50.0,[*cpsbreak*]*p* = 1.8e−12, *F*_1,4553_ = 66.9, *p* = 3.6e−16, *F*_1,4570_ = 10.7, *p* = [*cpsbreak*]0.0011, *F*_1,4559_ = 30.8, *p* = 3.1e−08, for target, masker, foreground, background noise, and entire acoustic scene decoders, respectively) and a significant effect of the end time of the overall integration window on the reconstructions accuracy of the target, masker, foreground, and acoustic scene envelopes (Figures 7C,D, *F*_3,4533_ = 119.5,[*cpsbreak*]*p* = 2.2e−16, *F*_3,4551_ = 16.7, *p* = 8.8e−11, *F*_3,4553_ = 40.5, *p* = 2.2e−16, *F*_3,4531_ = 213.9, *p* = 2e−16 for target, masker, foreground, and entire acoustic scene decoders, respectively), but not on the reconstruction accuracy of the background noise envelope (*F*_3,4533_ = 2.3, *p* = 0.075). We did not observe any significant interactions between the NR and the end time of the overall integration window for any of the decoders. The *post hoc* pairwise comparisons (see Figures 7C,D) showed similar trends as our *post hoc* pairwise comparisons results for late integration windows from section “Late Neural Responses.”

We further examined the relationship between late responses and overall responses. More precisely, we wondered if reconstruction accuracy of the target, masker, foreground, background noise and entire acoustic scene were higher for either EEG response windows (i.e., late or overall integration window). Two-way LMM ANOVAs fitted for the target, masker, foreground, background noise and entire acoustic scene decoders confirmed a significant effect for NR (*F*_1,9761_ = 65.7, *p* = 5.6e−16,[*cpsbreak*]*F*_1,9778_ = 106.2, *p* = 2e−16, *F*_1,9761_ = 112.2, *p* = 2e−16, *F*_1,9784_[*cpsbreak*] = 24.7, *p* = 7e−07, *F*_1,9769_ = 45.6, *p* = 1.6e−11 for target, masker, foreground, background noise and entire acoustic scene decoders, respectively), and a significant effect of the EEG response window duration for the entire acoustic scene and foreground decoders (*F*_1,9736_ = 66.5, *p* = 4e−16, *F*_1,9737_ = 4.4, *p* = 0.0356, respectively), but did not reveal a significant effect for EEG response windows for target, masker, and background noise decoders (*F*_1,9737_ = 1.07, *p* = 0.3205, *F*_1,9736_ = 0.8, *p* = 0.3583, *F*_1,9737_ = 0.8, *p* = 0.3728 for target, masker, and background noise decoders, respectively) and interaction (*F*_1,9737_ = 0.5, *p* = 0.4885, *F*_1,9736_ = 0.1, *p* = 0.7820, *F*_1,9736_ = 1.0, *p* = 0.3106, *F*_1,9737_ = 0.03, *p* = 0.8593, *F*_1,9736_ = 0.9, *p* = 0.3513 for target, masker, foreground, background noise, and entire acoustic scene decoders, respectively). This indicates that the envelopes of the target, masker, and background noise could be reconstructed with similar fidelity from late EEG responses only compared to the overall EEG responses which are often used in the literature.

### Noise Reduction Effects for Behavioral Performances

**FIGURE 8:**
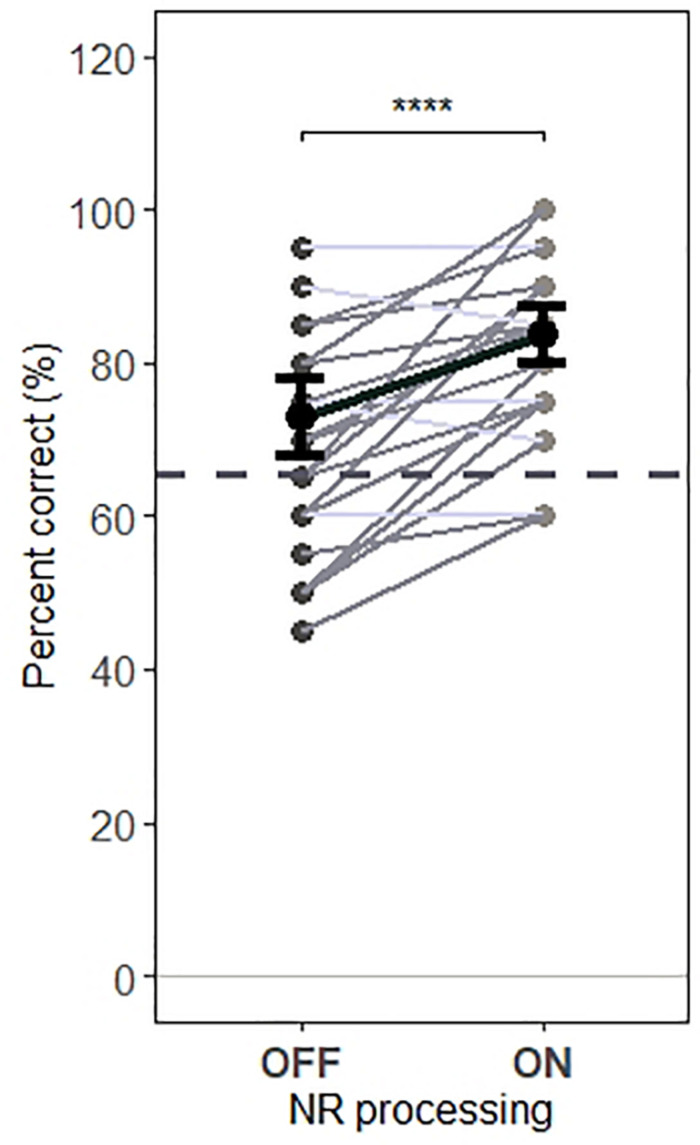
Behavioral performance for each condition. The NR scheme improves the behavioral performance. A significant main effect of NR scheme on behavioral performance was observed, indicating increased participants’ accuracy in responding to questions about target speech, when NR scheme was active. Gray dots indicate trial-averaged individual behavioral performance results, whereas black dots and error bars show the group (grand-average ± 1 between subject SEM) behavioral performance results. Each gray dot represents the behavioral performance results for a single participant, each horizontal line in gray denotes single participant (dark gray denotes increased accuracy and light gray denotes reduced accuracy when activating NR scheme) and each horizontal line in black depict prediction of linear mixed model. The dashed horizontal line in gray the behavioral performance level at which behavioral results are significantly greater than chance (65.625 %) based on a binomial test at the 5% significance level. The asterisk indicates significant differences (*p=0.05, **p = 0.01, ***p = 0.005, and ****p = 0.0001).

Behavioral performance results showed that participants were able to successfully follow the instructions and pay attention primarily to the target speech. Figure 8 shows the accuracy of participants in responding to questions about target speech for each testing condition (“NR OFF” and “NR ON”). In both conditions, participants performed well above chance levels for both NR conditions (73% correct and 84% correct for “NR OFF” and “NR ON” conditions, respectively). Furthermore, a one-way RM ANOVA revealed a significant main effect of NR (*F*_1,1279_ = 22.3, *p* = 2.5e−06), indicating that NR improved behavioral performances.

## Discussion

In this study we used envelope reconstruction to investigate the relationship between NR and cortical tracking of continuous speech at different stages of the auditory system. Envelope reconstruction accuracy (i.e., the correlation between the reconstructed stimulus envelope and the actual speech envelope) served as a measure of the fidelity of the cortical representation of that speech envelope. We found that reconstruction accuracy for the competing talkers and the background noise were strongly affected by the NR scheme. This was seen first in the early EEG responses, where the reconstruction accuracy of the foreground and entire acoustic scene was found to be more accurate with the NR on compared to off. Although we did not expect to find differential effects of NR for the target vs. masker speech in the early responses, the finding of higher reconstruction accuracy in the foreground did appear to be driven mainly by NR effects specifically for the target talker (Figure 4).

These findings were also confirmed in the analysis of the late EEG responses (Figure 5), where the reconstruction accuracy was higher for target and masker and lower for the background with the NR on compared to off, a finding that is consistent with our previous study (Alickovic et al., 2020). The results also revealed that the late EEG responses selectively represent the target talker with significantly higher fidelity than masker talkers, a finding that is consistent with previous studies using MEG (Ding and Simon, 2012a; Puvvada and Simon, 2017) and ECoG (Mesgarani and Chang, 2012; O’Sullivan et al., 2019).

### Representation of the Foreground and Entire Acoustic Scene in Early EEG Responses Is Affected by Noise Reduction

The results from our analysis confirm the significant effect of NR on cortical representation of the foreground and acoustic scene mixture in early EEG responses. We found that reconstruction accuracy for the foreground and the entire acoustic scene was higher in the early responses when the NR scheme was active compared to inactive (Figures 4A,B). Our findings extend recent ECoG results using NH listeners by O’Sullivan et al. (2019). Their data suggested that early responses in Heschl’s gyrus (primary areas of auditory cortex) support a rich representation of the acoustic scene mixture which may facilitate decoding and extraction of both target and masker talkers in later responses in higher-order cortical areas. Moreover, we here demonstrate that, in HI listeners, NR can contribute to speech processing in HI listeners by providing a richer representation (i.e., higher reconstruction accuracy) of the entire acoustic scene mixture and foreground as compared to inactive NR, thus potentially facilitating extraction and decoding of both target and masker talkers in later EEG responses.

In addition, recent MEG evidence by Brodbeck et al. (2020) suggests effect of selective attention on early responses. In other words, early responses, in addition to representing an acoustic scene mixture, are also sensitive to neural processes that may relate to active reconstruction of acoustic features that could originate from either stream. We may also expect the early effect of attention from animal studies which have previously shown task-dependent modulations of responses in the primary auditory cortex (Fritz et al., 2003; Atiani et al., 2009). We therefore extended our analysis to explicitly examine the representation of individual objects (i.e., target, masker and background noise) during early EEG responses in HI listeners. Our results revealed that reconstruction accuracy of the target envelope (Figure 4C), but not for the masker talker envelope (Figure 4D) and background noise (Figure 4E) envelope, was significantly higher when the NR scheme was active as compared to inactive. The results suggest that our first finding of higher reconstruction accuracy for the foreground and entire acoustic scene may be driven by better reconstruction accuracy selectively for the target talker, even in the early EEG responses. This is a tantalizing finding that should be followed up with more detailed investigations of the representations of target vs. masker talkers in early responses.

Furthermore, we investigated how well the target source could be classified from early EEG responses by analyzing classification accuracy. Based on these aforementioned studies (Puvvada and Simon, 2017; O’Sullivan et al., 2019), we did not expect a large effect of attention in early responses, which would result in classification accuracy (i.e., the differentiation between target and masker talkers) not significantly greater than chance. Our results revealed that the classification accuracy was below the chance level when the NR scheme was turned off but was significantly higher and above chance when the NR was on. Taken together, classification analysis results suggested a significant effect of NR and weak but significant effect of attention on early EEG responses. Overall, as hypothesized (H1), our results suggested that the active NR provided a richer representation (i.e., higher reconstruction accuracy) of the foreground and acoustic scene mixture when it is active as compared to inactive, and that this increase may be driven by NR-related improvements in reconstruction accuracy specifically for the target talker.

### Representation of the Individual Elements in Late EEG Responses Is Affected by Noise Reduction

We showed that the NR scheme enhanced reconstruction accuracy for the target (Figure 5C) and masker (Figure 5D) talkers in the front hemifield and reduced reconstruction accuracy for the noise in the background (Figure 5E) in late EEG responses. Furthermore, as a complementary finding, we showed that the NR scheme enhanced the representation of the foreground (Figure 5B), while the net sum of the entire acoustic scene (Figure 5A) was not enhanced in late EEG responses. This approach was based on findings in recent studies suggesting that responses to the acoustic envelope reflected processing of the acoustic mixture. Neither one of these effects are surprising as the relative benefits of SNR (Das et al., 2018; Decruy et al., 2020) and NR on cortical representations of competing talkers are well established (Alickovic et al., 2020; Lunner et al., 2020). However, previous studies did not distinguish between early and late responses, whereas our analysis assessed cortical representations of individual elements in early, late, and overall EEG responses, revealing a significant effect of NR on speech representation in late EEG responses.

In a recent study similar to ours, Vanheusden et al. (2020) compared cortical responses to speech in HI listeners wearing HAs (with the NR scheme inactive) and without HAs. They reported that entrainment of alpha, theta and wideband EEG responses to the envelope of clean speech presented at an audible level was not affected by HAs. A likely explanation for the lack of difference in these entrainment responses with the HA on vs. off is that Vanheusden et al. (2020) had only one talker presented at a comparatively high intensity, well audible for the participants, even in the unaided condition. Thus, there may have been minimal perceptual differences between the conditions. In contrast, the results in the current study were focused on differences related to NR, and our stimulus setup included noise sources that would likely be strongly attenuated by the NR algorithm, thus leading to potentially large perceptual differences between the conditions. We also used a selective attention task, and reconstruction accuracy has been previously shown to be larger to attended vs. unattended speech (Alickovic et al., 2020; Fuglsang et al., 2020). Moreover, we found a better behavioral performance in terms of recalling the content of the attended speech when activating NR, consistent with NR resulting in more accurate late representation of the target talker.

### Late EEG Responses Are Affected by Selective Attention

We found significantly better reconstruction accuracy and classification accuracy for the target compared to the masker talker in the late EEG responses, suggesting a separated representation of the target talker. These findings are consistent with previous studies using MEG (Puvvada and Simon, 2017; Puschmann et al., 2019; Brodbeck et al., 2020) and ECoG (Mesgarani and Chang, 2012; Zion Golumbic et al., 2013; O’Sullivan et al., 2019) in NH listeners showing that the late responses in higher-order auditory area involved in speech processing primarily track the target stream. In summary, these results confirm our hypothesis H2–that the late EEG responses in HI listeners selectively represent the target talker with significantly higher fidelity than the masker talker and background noise.

### Relationships Among Early Responses, Late Responses, Overall Responses, and Noise Reduction

In line with previous studies (Puvvada and Simon, 2017; O’Sullivan et al., 2019; Brodbeck et al., 2020; Norman-Haignere et al., 2020), our results showed significant differences in reconstruction accuracy between early and late integration windows, indicating enhanced envelope representation and significant effect of attention in the late EEG responses. This can be seen by comparing the reconstruction values across Figures 4, 5, or observing the data replotted in Figure 6.

One major difference between our study and previous work is that we have separately analyzed early, late and overall EEG responses, whereas the majority of previous studies did not distinguish between these time windows [e.g., Das et al. (2018), Presacco et al. (2019), Broderick et al. (2020a)]. Our results revealed a significant effect of NR on reconstruction accuracy across both EEG response windows (late and overall) for each envelope type, and a significant effect of EEG response window on reconstruction accuracy for the acoustic scene and foreground decoders, but did not reveal a significant effect of EEG response windows for target, masker and background noise decoders, indicating that the target, masker, and background noise envelopes can be reconstructed with similar fidelity from late EEG responses only, as from the overall EEG responses.

Furthermore, speech processing is thought to follow a serial and hierarchical organization in the brain. Therefore, hypothesized experimental effects can be found in the EEG at different latencies depending on the type of task or experimental manipulation. In addition, the analytic methods used to extract the EEG response perform differently depending on the duration of the analysis window used (Bouchard et al., 2013; Presacco et al., 2016, 2019; Brodbeck et al., 2018; Broderick et al., 2020b; Norman-Haignere et al., 2020). A few recent studies investigating differences in cortical tracking of speech across different groups of listeners reported the decline of reconstruction accuracy with narrowing the time window (from 500 ms down to 150 ms) in older adults, meaning that older adults require more time to process the information in the speech signal (Presacco et al., 2016, 2019). Our integration window analysis results extended these findings, and demonstrate that both the NR scheme and the time window duration have significant effects on the strengths of the cortical representation of the target talker and masker talkers, with strong reconstruction accuracies seen for window durations up to 350 and 250 ms respectively. Interestingly, our analysis of the background noise envelope reconstruction showed that the NR scheme but not the time window duration had a significant effect on the strength of the cortical representation. It would be interesting, in future work, to determine the relative contribution of each time latency to representation of different elements of acoustic scene using forward modeling approach [e.g., Fiedler et al. (2019)].

Taken together, the results validate our stimulus reconstruction approach as suitable to investigate and potentially compare the degree to which listeners are able to encode different sound sources in complex listening environments. By examining the cortical representation of individual elements in early and late EEG responses and how these representations are affected by a NR scheme, our study takes an important step toward determining the benefits of a NR scheme on the representation of complex multi-talker auditory scenes in different areas of the auditory cortex in participants with impaired hearing.

## Conclusion

We studied how the NR scheme in commercial HAs affected the representation of complex multi-talker auditory scenes. We found that neural representations of target and masker talkers located in front as well as the background were significantly affected by the NR scheme. Specifically, we found that the use of NR scheme contributed to the enhancement of the foreground and of the entire acoustic scene in the early EEG responses (thought to be dominated by primary-like areas), and that this enhancement was driven by better representation of the target speech. Furthermore, the target talker was selectively represented in late responses thought to have sources in higher-order non-primary areas. We also found that use of the NR scheme resulted in enhanced representations of the target and masker speech in the foreground and a suppressed representation of the noise in the background in late responses. We found significant effect of EEG time window on the strengths of the cortical representation of the target and masker. Finally, we found that the envelopes of the target, masker, and background noise could be reconstructed with similar fidelity from late EEG responses only compared to the overall EEG responses. Together, our analyses of the early and late responses obtained from HI listeners support the existing view of hierarchical processing in the auditory cortex. Our findings demonstrate the benefits of a NR scheme on the representation of complex multi-talker auditory scenes in different areas of the auditory cortex in participants with impaired hearing.

## Data Availability Statement

There are ethical restrictions on sharing the data set. The consent given by participants at the outset of this study did not explicitly detail sharing of the data in any format; this limitation is keeping with EU General Data Protection Regulation, and is imposed by the Research Ethics Committees of the Capital Region of Denmark. Due to this regulation and the way data was collected with low number of participants, it is not possible to fully anonymize the dataset and hence cannot be shared. As a non-author contact point, data requests can be sent to Claus Nielsen, Eriksholm research operations manager at clni@eriksholm.com.

## Ethics Statement

The studies involving human participants were reviewed and approved by Ethics Committee for the Capital Region of Denmark (journal number H-1-2011-033). The patients/participants provided their written informed consent to participate in this study.

## Author Contributions

EA, EN, LF, SS, HI-B, and CG contributed to study concept, hypothesis generation, design of the experiment, interpreted the data, read the manuscript, and provided critical revision. EA, CG, and research clinicians recorded the data. EA analyzed the data and drafted the manuscript. All authors contributed to the article and approved the submitted version.

## Conflict of Interest

The authors declare that the research was conducted in the absence of any commercial or financial relationships that could be construed as a potential conflict of interest.
